# Ferulic Acid Esterase-Producing Inoculant Improves Fiber Degradation and Modulates Microbial Diversity in Corn Bran Silage and Whole-Plant Corn Silage

**DOI:** 10.3390/microorganisms13112439

**Published:** 2025-10-24

**Authors:** Yang Yu, Xiaojun Guo, Haoer Li, Chen Yu, Hao Liu, Wei Guo

**Affiliations:** College of Life Sciences, Hebei Agricultural University, No. 289 Lingyusi Street, Baoding 071000, China; 20242050080@pgs.hebau.edu.cn (Y.Y.);

**Keywords:** *Bacillus amyloliquefaciens*, cellulose degradation, ferulic acid esterase, microbial diversity, nutritional quality, whole-plant corn silage

## Abstract

Ferulic acid esterase (FAE) catalyzes the hydrolysis of the feruloyl ester bond in lignocellulose, exposing cellulose. The objective of this research was to examine the impacts of *Bacillus amyloliquefaciens* A30 producing FAE on the fermentation quality, fiber degradation, enzyme activity and microbial diversity of corn bran silage and whole-plant corn silage. The experimental treatments were as follows: control (CK), cellulase (CEL), strain A30 (A30) and CEL + A30. Corn bran and whole-plant corn were ensiled for 14 d and 60 d, respectively. The results showed that all additive treatments effectively reduced the pH, neutral detergent fiber, acid detergent fiber and cellulose contents of both corn bran silage and whole-plant corn silage in comparison with control, with CEL + A30 group performing the best effects. Meanwhile, higher FAE activity was detected in A30 and CEL + A30 groups during ensiling. Furthermore, the supplementation of A30 increased the degradation ratio of NDF, ADF, ADL, and cellulose of corn bran silage and whole-plant corn silage. Additionally, treatments with A30 and CEL + A30 increased the abundance of *Lactobacillus*, and reduced the proportion of pathogenic genera, including *Acinetobacter*, *Enterobacter*, and *Sphingobacterium*. In conclusion, the application of A30 may effectively promote fiber degradation and the stability of microecological system for corn silage.

## 1. Introduction

Whole-plant corn is the type of silage that is used most widely, having low buffering capacity and a high content of water-soluble carbohydrates [1]. Cellulose and hemicellulose, as key structural constituents of corn stover, represent the most abundant carbohydrate sources for ruminants. Nevertheless, lignification imposes a significant constraint on the bioavailability of these carbohydrates. Currently, the supplementation of cellulases or cellulose-degrading strains constitutes the primary strategy to hydrolyze cellulose into small-molecular-weight compounds, thereby enhancing their digestive utilization by livestock [2,3]. As a by-product of corn, corn bran is the outer layer separated during the corn kernel processing. It is rich in various active substances such as cellulose, corn fiber oil, and feruloyl oligosaccharides. During the wet processing method, the soaking liquid of corn is sprayed onto the corn bran and then dried. After spraying, the protein content of corn bran increased. Therefore, sprayed corn brans have the potential to be widely used as feed raw materials in animal husbandry [4]. However, the high cellulose content limits their effective utilization.

Hemicellulose and lignin surround cellulose, creating a dense and resistant structure that impedes enzymatic hydrolysis. Ferulic acid enables the formation of cross-links between lignin and lignin, lignin and hemicellulose, and hemicellulose and hemicellulose through feruloyl ester bonds, which further enhances the mechanical robustness of the cell wall [5]. Additionally, the cross-linking between hemicellulose and lignin mediated by ferulic acid is generally acknowledged as a crucial factor that hinders the degradation of polysaccharides by microbial enzymes or exogenous fibrolytic enzymes [6]. Concurrently, this cross-linking also reduces the fermentation efficiency of plant cell walls in the rumen. Ferulic acid esterase (FAE) catalyzes the hydrolysis of feruloyl ester bonds in lignocellulose, thereby exposing cellulose. This exposure renders cellulose more susceptible to digestion within the cell wall and enhances its degradation rate [7,8]. Most FAEs are derived from extracellular secretions of microorganisms, including filamentous fungi and bacteria [9]. However, the industrial production of FAEs is associated with high costs, rendering them unsuitable for direct application as feed fermentation additives. Accordingly, recent research has increasingly centered on the direct application of FAE-producing strains.

Inoculation with FAE-producing strains during silage fermentation can enhance the degradation of lignocellulose in feedstuffs. Andrada et al. [10] conducted a review on the ensiling effects of 17 previously reported FAE-producing lactic acid bacteria (LAB) strains. Their findings revealed that, relative to the uninoculated silage controls, a 1.3–6.6% reduction in neutral or acid detergent fiber content represented the most prevalent outcome when significant treatment effects were observed. At present, *Bacillus* species are widely used for silage. *Bacillus*, a facultative anaerobic bacterium, can consume oxygen sources to create an anaerobic environment, which stimulates the growth of lactic acid bacteria and the acidification of silage, thus suppressing the growth of spoilage microbes and minimizing nutrient loss [11]. Therefore, *Bacillus* can optimize the bacterial community by producing bacteriocin, which inhibits pathogens, such as yeasts, *Escherichia coli*, *Salmonella*, *Listeria*, and *Salmonella pullorum* [12]. Moreover, it has been found to improve feed digestibility by secreting α-amylase, xylanase, and protease [13]. Manhar et al. isolated *Bacillus amyloliquefaciens* from traditional fermented soybean, which showed potential probiotic characteristics as well as a significant cellulolytic activity in vitro [14]. As a commonly used silage bacteria agent, it can increase the concentration of lactic acid and improve the quality of whole-plant corn silage [15].

In our previous study, strain *Bacillus amyloliquefaciens* A30 was isolated from the feces of *Potosia brevitarsis*, which could effectively degrade ferulic acid ester, with an FAE activity of 177.0 U/L. However, whether this strain can exert similar lignocellulose degradation effects in silage systems remains unclear. In the present study, *Bacillus amyloliquefaciens* A30 was employed for ensiling corn bran and whole-plant corn. It was hypothesized that *Bacillus amyloliquefaciens* A30 can significantly reduce the fiber content. The objective was to evaluate fiber degradation, analyze microbiota changes, and assess the strain’s application potential. This approach provides a theoretical foundation for practical feed production and livestock industry advancement.

## 2. Materials and Methods

### 2.1. Raw Materials and Silage Additives

At the dough stage on 5 October, whole-plant corn was harvested from the experimental base of Hebei Agricultural University in Baoding, China (latitude 38.82° N, longitude 115.44° E, elevation 20 m), and then chopped into filaments 1–2 cm in length with a crop cutter. Then, prior to ensiling, half of the chopped whole corn was wilted until it reached an approximate dry matter (DM) content of 350 g/kg of fresh weight (FW). Meanwhile, corn kernels were manually stripped from the remaining crop materials. These kernels were soaked in 0.12% sulfurous acid at 50 °C for 26 h, after which the concentrated corn slurry was collected. Subsequently, the sulfurous acid-soaked corn kernels were crushed using a crusher until the corn germs were completely separated. Following the removal of the germs, the remaining residue was subjected to fiber separation to isolate corn bran. The isolated corn bran was then mixed with the previously collected concentrated corn slurry, and the mixture was dried. The dry matter content of the dried corn bran was determined to be 924 g/kg. Thereafter, 1050 mL of distilled water was added to 1000 g corn bran, and the mixture was thoroughly homogenized to obtain a final material with a dry matter content of approximately 450 g/kg FW. The detailed nutrient compositions of corn bran and whole-plant corn are presented in Table 1.

FAE-producing strain *Bacillus amyloliquefaciens* A30 and commercial cellulase provided by Xin Dayang Neiqiu Biotechnology Co., Ltd. (Baoding, China) were employed as silage additives. Strain A30, which exhibits FAE-producing activity, was isolated and screened from the feces of *Potosia brevitarsis* using the agar-based screening method described by Donaghy et al. [16]. It was preserved at −80 °C in nutrient broth supplemented with 30% (*v*/*v*) glycerol. Commercial cellulase, supplied as a lyophilized powder, was stored at 4 °C. According to the manufacturer’s instructions, the cellulase activity of the powder was over 10,000 U/g. It primarily contained a patented mixture of plant cell wall degrading enzymes, such as *β*-glucanase, *α*-arabinase, *α*-galactosidase, *β*-galactosidase, and *β*-xylanase.

### 2.2. Ensiling and Sampling

The corn bran silage and whole-plant corn silage were divided into 4 treatments, respectively: (1) control (CK); (2) cellulase (CEL, cellulase 3 U/g FW); (3) *Bacillus amyloliquefaciens* A30 (A30, 5 × 10^6^ CFU/g FW); and (4) cellulase combined with *Bacillus amyloliquefaciens* A30 (CEL + A30, cellulase 3 U/g FW and A30 5 × 10^6^ CFU/g FW). Prior to ensiling, strain A30 was thawed overnight at 4 °C and propagated twice in nutrient broth medium before use. After incubation, A30 was assayed for viable cell counts, then centrifuged and resuspended in sterile distilled water to a viable count of 5 × 10^8^ CFU/mL. The 10 L prepared bacterial suspension of *Bacillus amyloliquefaciens* A30 was uniformly sprayed onto 1 t of silage raw material, with a final additive dosage of the A30 strain in the silage at 5 × 10^6^ CFU/g FW. After thorough mixing, the mixture was sealed for fermentation. Similarly, commercial cellulase was dissolved in sterile distilled water. The additives were then uniformly sprayed by a sprayer and thoroughly homogenized with silage. The material in the control group was sprayed with an equal volume of distilled water. Subsequently, all treated corn bran was compacted into plastic bottles (volume: 500 mL) with paraffin sealing and an initial density of 0.78 g/cm^3^. The whole-plant corn silage was compacted into silage bags and vacuum sealed with a vacuum packaging machine at an initial density of 0.8 g/cm^3^. Corn bran and whole-plant corn were ensiled for 14 d and 60 d, respectively, under a controlled room temperature of 25 ± 0.2 °C. Sampling was performed using the five-point sampling method: corn bran silage was sampled after 0, 1, 3, 6, 10, and 14 days of ensiling, and whole-plant corn silage was sampled after 0, 7, 14, 30, 45, and 60 days of ensiling, with three replicates collected for each sample. The above samples were used to determine enzyme activity. Corn bran silage samples from 0 d and 14 d and whole-plant corn silage from 0 d and 60 d were used to assess fermentation profile, lignocellulose degradation, and microbial diversity.

### 2.3. Analytical Methods

#### 2.3.1. Fermentation Profile

For each sample, 10 g of FW material was homogenized with 100 mL of sterile distilled water. The mixture was allowed to stand undisturbed for 20 min, followed by shaking at 180 rpm on a reciprocating shaker for another 20 min. A calibrated electronic pH meter was employed to measure the pH value of the obtained supernatant. Meanwhile, 10 g sample was pretreated in 1% (*v*/*v*) aqueous sulfuric acid (100 g/L) in a 250 mL Erlenmeyer flask, reacting at 120 °C for 2 h in a pressure sterilizer. Then, 1 mL filtrate was centrifuged (14,000 rpm, 4 °C, 5 min). The resulting supernatant was re-filtered and diluted 1:1 in 0.5% meta-phosphoric acid (Sigma-Aldrich, St. Louis, MO, USA). A 20 μL of this solution was collected to detect lactic acid and acetic acid content by HPLC [17,18].

#### 2.3.2. Nutritional Quality and Fiber Degradation Analyses

A 10 g of FW from each sample was dried in a forced-air drying oven at 105 °C to constant weight. The DM was calculated as the percentage of the sample mass remaining after drying relative to the initial fresh weight. Two grams of oven-dried sample was prepared for the determination of crude protein (CP) and acid-soluble protein (ASP) content. Crude protein was determined directly by the Kjeldahl method [19]. For ASP, 3 g of dried sample was soaked in 25 mL of 15% trichloroacetic acid for 4 h. The supernatant was centrifuged at 10,000 rpm for 5 min and then determined by Kjeldahl method. The fat (Fat) content was determined by the method of Terré et al. [20]. A fiber analyzer (Model A2000i, Ankom Technology, Macedon, NY, USA) was used to analyze neutral detergent fiber (NDF), acid detergent fiber (ADF), and acid detergent lignin (ADL), with the analysis procedure referring to the method established by Van Soest et al. [21]. Hemicellulose content was estimated by subtracting the ADF value from the NDF value; likewise, cellulose content was estimated by subtracting the ADL value from the ADF value. The fiber degradation ratio was calculated as follows: the difference between the fiber content at the initiation of ensiling and that at the termination of ensiling was divided by the fiber content at the initiation of ensiling, then multiplied by 100%.

#### 2.3.3. Determination of Microorganism Populations

Microbial populations of bacteria, LAB, and molds were tested by gradient dilution method using nutrient agar, de Man, Rogosa and Sharpe agar, and potato dextrose agar, respectively.

#### 2.3.4. Determination of FAE and Cellulase Activity

A 10 g sample was homogenized with 50 mL of 0.1 mol/L sodium acetate–acetic acid buffer (pH 5.0). The resulting mixture was transferred to a shaking incubator at 37 °C with constant agitation at 180 rpm for 30 min. Following incubation, the mixture was centrifuged at 10,000 rpm for 15 min at 4 °C to separate the solid residues from the liquid phase. The supernatant was carefully collected and filtered through a 0.22 μm aqueous-phase filter membrane, thus obtaining the crude enzyme extract. The crude enzyme extract was subsequently used for the determination of ferulic acid esterase activity and cellulase activity, respectively. The enzyme activity of FAEs was determined by HPLC. Cellulase activity was assayed via the dinitrosalicylic acid method. Specifically, the reducing sugars produced were measured at a wavelength of 540 nm using a UV spectrophotometer (UV-1600, Shanghai Mapada Instrument Co., Ltd., Shanghai, China).

#### 2.3.5. Analysis of the Microbial Diversity

The methods for analyzing microbial diversity were referenced by Guo et al. [22]. In accordance with the kit instructions, a DNA extraction kit (Tiangen Biotech Co., Ltd., Beijing, China) was used to extract the total genomic DNA of the microbes. The quality and concentration of the DNA were measured using an ultra-micro UV spectrophotometer and 1% agarose gel electrophoresis. The V3-V4 region of the bacterial 16S rRNA gene was amplified using primers 338F (5′-ACTCCTACGGGAGGCAGCAG-3′) and 806R (5′-GGACTACHVGGGTWTCTAAT-3′). PCR products of the same samples were mixed and retrieved using 2% agarose gel. Then, they were purified with an AxyPrep DNA Gel Extraction Kit (Axygen Biosciences, Union City, CA, USA), detected by 2% agarose gel electrophoresis, and quantified with a Quantus Fluorometer (Promega, Madison, WI, USA). The NextFlex Rapid DNA-Seq Library Construction Kit (Bioo Scientific, Austin, TX, USA) was employed for library construction. Subsequently, pooled samples were sequenced on the HiSeq 2500 platform (Illumina, San Diego, CA, USA) at Shanghai Majorbio Bio-pharm Technology Co., Ltd. (Shanghai, China) according to standard protocols.

OTU clustering was carried out on the sequences at a similarity level of 97%. According to the *α*-diversity index, the microbial diversity in the silage was analyzed, including the Shannon index, Simpson index, Chao index, and Ace index. Through principal coordinates analysis (PCA), the original complex data was simplified to uncover the similarities and differences between samples concealed behind the complex data. To explore the relationship between the microflora and silage quality of corn bran silage and whole-plant corn silage, correlation analysis was performed and a heatmap was generated.

### 2.4. Statistical Analysis

After normal distribution examination with the Shapiro–Wilk test and variance homogeneity examination, two-way analysis of variance was conducted utilizing the general linear model module in SPSS 27.0 Statistics. When the main effect of either factor or the interaction effect between the two factors was statistically significant (*p* < 0.05), pairwise comparisons were performed based on estimated marginal means, with the Bonferroni correction applied to account for multiple comparisons. Data were presented as “mean ± SD”.

## 3. Results

### 3.1. Fermentation Characteristics

Inoculation with A30 had significant effects on pH and acetic acid content of corn bran (Table 2). In the enzyme treatments, the pH was lower and acetic acid content was higher in the inoculation group than the non-inoculation group (*p* < 0.05). Adding A30 enhanced the pH value of whole-plant corn but did not significantly affect the lactic acid and acetic acid content. (Table 3).

### 3.2. Nutritional Quality and Fiber Degradation

In the corn bran silage, inoculation with A30 had significant effects on CP, ASP, NDF, ADF, and cellulose contents. As shown in Table 4, the addition of cellulase, A30, and their complex significantly reduced NDF, ADF, and cellulose contents of corn bran silage (*p* < 0.05). Meanwhile, corn bran silage treated with the complex of strain A30 and cellulase had the lowest NDF, ADF, and cellulose contents after 14 days fermentation. Moreover, the A30 group had higher hemicellulose content than other groups (*p* < 0.05).

In the whole-plant corn silage, inoculation with A30 had significant effects on NDF, ADF, hemicellulose, and cellulose contents. As shown in Table 5, in comparison with control group, inoculation with cellulase, A30 and their complex all decreased the NDF, ADF, hemicellulose, and cellulose contents significantly (*p* < 0.05).

Fiber degradation ratio of corn bran and whole-plant corn is shown in Table 6. The separated addition of A30 and cellulase showed higher degradation ratio of NDF, ADF, ADL, and cellulose than the control of corn bran silage. And supplementation of their complex further increased those degradation ratios. For the whole-plant corn silage, adding A30 or cellulase alone enhanced all the fiber degradation ratio. Similarly, the CEL + A30 group showed significantly higher fiber degradation ratio than A30 and CEL groups, except ADL.

### 3.3. Microorganism Quantity

Inoculation with A30 had no effects on Bacteria, LAB, and Mold number of corn bran (Table 7). Inoculation with A30 improved Bacteria number (*p* < 0.05) of whole-plant corn (Table 8), whereas had no effects on LAB and Mold number.

### 3.4. Enzyme Activity

**Figure 1:**
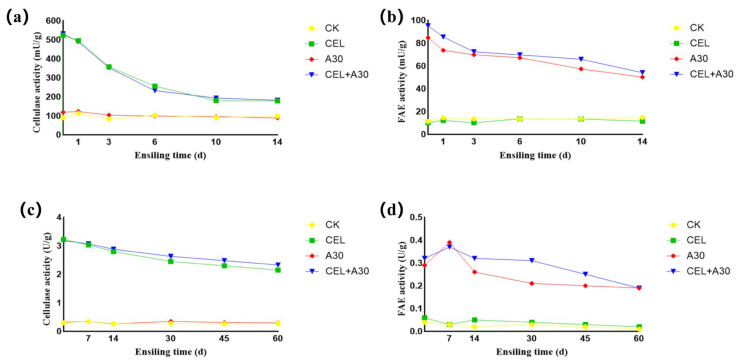
Dynamic enzyme activity of corn bran silage and whole-plant corn silage. (**a**) Cellulase activity of corn bran silage for 14 days. (**b**) Ferulic acid esterase activity of corn bran silage for 14 days. (**c**) Cellulase activity of whole-plant corn silage for 60 days. (**d**) Ferulic acid esterase activity of whole-plant corn silage for 60 days. CK, silage with no additive; CEL, silage treated with cellulase; A30, silage treated with *Bacillus amyloliquefaciens* A30; CEL + A30, silage treated with cellulase and *Bacillus amyloliquefaciens* A30.

Adding strain A30 alone cannot change the cellulase activity of corn bran silage and whole-plant corn silage (Figure 1a,c), whereas the inoculation of cellulase and its mixture with strain A30 enhanced the cellulase activity of both corn bran silage and whole-plant corn silage. Moreover, their levels showed a downward trend during the silage fermentation stage. Similarly, the application of A30 and its mixture with cellulase improved the FAE enzyme activity of both corn bran silage and whole-plant corn silage (Figure 1b,d). And their activities were reduced during ensiling.

### 3.5. Microbial Diversity

**Figure 2:**
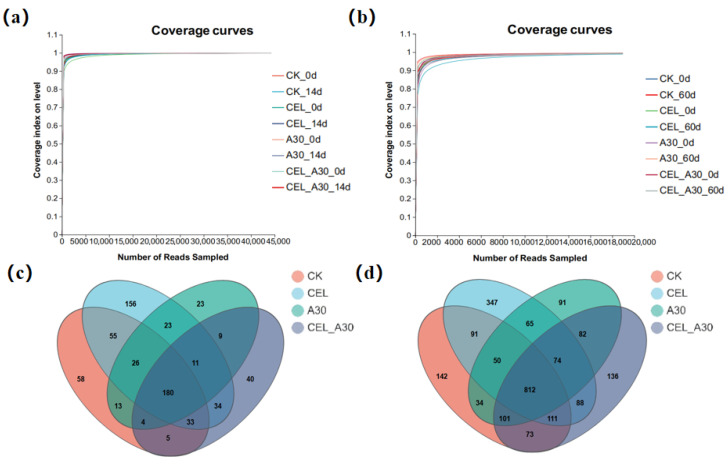
Dilution curves and Venn diagram. (**a**) Dilution curves of corn bran ensiled for 14 d; (**b**) Dilution curves of whole-plant corn ensiling for 60 d. (**c**) Venn diagram of bacterial OTUs in corn bran silage. (**d**) Venn diagram of bacterial OTUs in whole-plant corn silage. CK, silage with no additive; CEL, silage treated with cellulase; A30, silage treated with *Bacillus amyloliquefaciens* A30; CEL + A30, silage treated with cellulase and *Bacillus amyloliquefaciens* A30.

The dilution curves (Figure 2) were nearly flat, suggesting that the quantity of data from this sequencing satisfies the analysis need. At this sequencing depth, the coverage index of each sample stabilized and reached over 0.99, suggesting that the sequencing amount could cover most of the species in the samples and the sequencing outcomes were trustworthy (Table 9 and Table 10). As shown in Figure 2c, the total number of OTU species shared by the four treatment groups of corn bran silage was 180. Venn analysis found 812 shared OTUs among different whole-plant corn silage groups. And the number of OTUs unique to CK, CEL, A30, and CEL + A30 was 142, 347, 91, and 136, respectively (Figure 2d).

Before ensiling, the alpha diversity of bacteria in corn bran silage and whole-plant corn silage did not show obvious differences in the abundance of the bacterial community (Table 9 and Table 10). After ensiling, the Shannon index was significantly lower (*p* < 0.05), and the Simpson index was significantly higher (*p* < 0.05) in the CEL + A30 group compared to the CK group, indicating there was a decrease in bacterial community diversity of corn bran silage and whole-plant corn silage. Meanwhile, Chao index and Ace index of CEL + A30 group were significantly lower (*p* < 0.05) than CK group, suggesting a decrease in bacterial community richness of corn bran silage and whole-plant corn silage.

**Figure 3:**
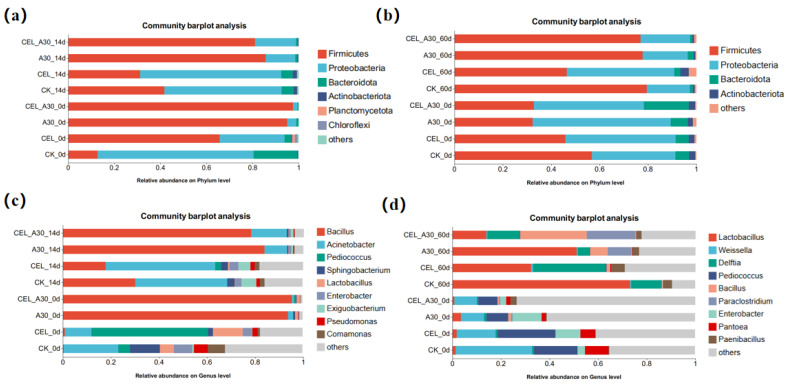
Relative abundance of bacteria in corn bran silage and whole-plant corn silage at phylum and genus levels. (**a**) Relative abundance of bacteria in corn bran silage at the phylum level. (**b**) Relative abundance of bacteria in whole-plant corn silage at the phylum level. (**c**) Relative abundance of bacteria in corn bran silage at the genus level. (**d**) Relative abundance of bacteria in whole-plant corn silage at the genus level. CK, silage with no additive; CEL, silage treated with cellulase; A30, silage treated with *Bacillus amyloliquefaciens* A30; CEL + A30, silage treated with cellulase and *Bacillus amyloliquefaciens* A30.

For corn bran silage, the most abundant bacterial phyla were Proteobacteria, Bacteroidota, and Firmicutes (Figure 3a). In comparison with CK, Firmicutes abundance was significantly higher (*p* < 0.05) in the A30 group. Meanwhile, the treatment with both cellulase and strain A30 significantly enhanced Firmicutes abundance compared with inoculation of cellulase alone. With the process of fermentation, the abundance of *Lactobacillus* in the A30 and CEL + A30 groups significantly increased, and *Bacillus* abundance decreased (Figure 3c).

For whole-plant corn silage, the abundance of Firmicutes significantly increased after ensiling compared to before, with absolute dominance in the A30 and CEL + A30 groups (Figure 3b). At the same time, the abundance of Proteobacteria and Bacteroidota decreased significantly. After 60 days of ensiling, the most prevalent bacterial genera in the CEL + A30 group were *Lactobacillus*, *Delftia*, *Bacillus*, and *Paraclostridium* (Figure 3d). In addition, the abundance of *Bacillus* significantly increased, and the abundance of *Enterobacter* and *Pantoea* decreased after 60 d of ensiling.

**Figure 4:**
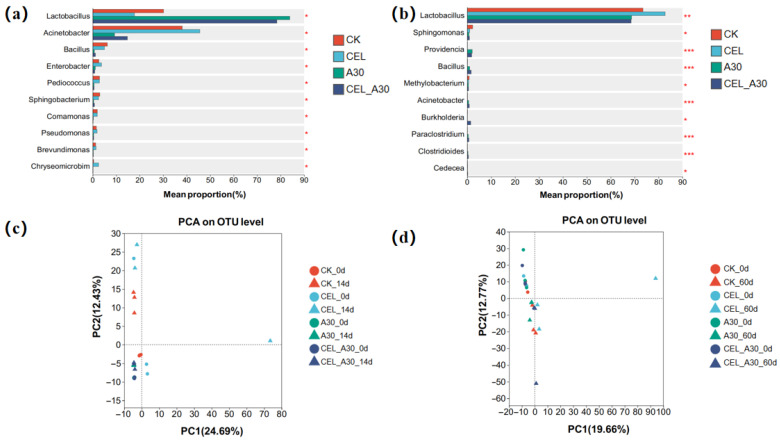
Comparison of different bacteria after ensiling using Tukey’s test and PCA analysis. (**a**) Comparison of different bacteria in corn bran silage. (**b**) Comparison of different bacteria in whole-plant corn silage. (**c**) PCA analysis of corn bran silage. (**d**) PCA analysis of whole-plant corn silage. “*”, “**” and ‘‘***” represent *p* ≤ 0.05, *p* ≤ 0.01 and *p* ≤ 0.001, respectively. CK, silage with no additive; CEL, silage treated with cellulase; A30, silage treated with *Bacillus amyloliquefaciens* A30; CEL + A30, silage treated with cellulase and *Bacillus amyloliquefaciens* A30.

After ensiling, the abundance of *Lactobacillus* in corn bran silage and the abundance of *Bacillus* of whole-plant corn silage were significantly higher in the A30 and CEL + A30 groups than CK and CEL groups (Figure 4a,b). Similarly, the abundance of *Providencia*, *Clostridioides* and *Paraclostridium* in whole-plant corn silage was significantly enhanced (*p* < 0.05, Figure 4b). In the corn bran silage, the abundance of *Acinetobacter*, *Enterobacter*, and *Sphingobacterium* was lower in the treatments with A30 and its combination with cellulase than those treatments with cellulase and without any addition (*p* < 0.05, Figure 4a). The results of PCA implied that all the groups significantly clustered apart from one another, suggesting that the bacterial assemblages varied significantly among all the groups (Figure 4c,d).

**Figure 5:**
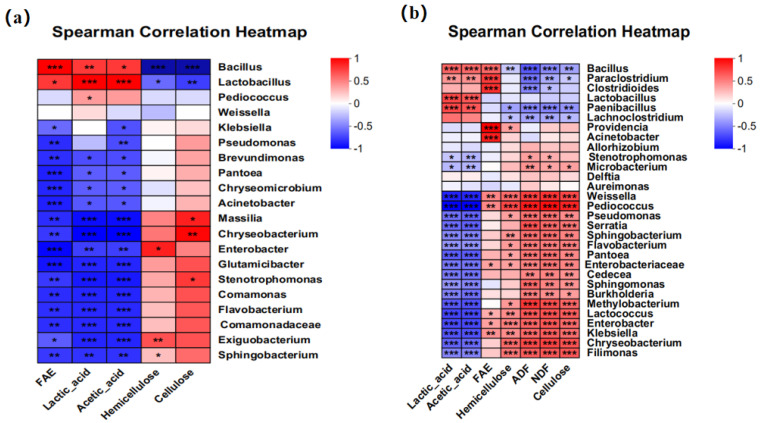
Correlation analysis between bacterial genera and fermentation quality indices in corn bran silage (**a**) and whole-plant corn silage (**b**). “*”, “**” and ‘‘***” represent *p* ≤ 0.05, *p* ≤ 0.01 and *p* ≤ 0.001, respectively. FAE, ferulic acid esterase activity; NDF, neutral detergent fiber; ADF, acid detergent fiber.

The Pearson correlation analysis was performed between fermentation quality and bacterial communities. The results showed that *Bacillus* in corn bran silage and whole-plant corn silage was positively correlated with FAE activity, contents of lactic acid and acetic acid, while it was significantly negatively correlated with both hemicellulose and cellulose contents (Figure 5). In addition, *Lactobacillus* was also positively correlated with the contents of lactic acid and acetic acid. In addition, structural carbohydrate contents, including NDF, ADF, hemicellulose, and cellulose, were significantly negatively correlated with *Bacillus*, *Paenibacillus*, *Clostridium*, and *Paraclostridium* in whole-plant corn silage. And FAE activity was significantly positively correlated with *Providencia*, *Bacteroides*, and *Clostridium*.

## 4. Discussion

The pH value serves as a crucial indicator for assessing fermentation success and is also commonly recognized as a significant index mirroring microbial activity during ensiling [23]. In corn bran silage, the co-treatment of cellulase with *Bacillus amyloliquefaciens* A30 reduced pH value by increasing acetic acid content. This is consistent with the findings of Li et al., who reported a significant increase in LA and acetic acid levels in silage, accompanied by a reduction in soluble carbohydrate content [24]. This phenomenon can be attributed to cellulose degradation, a process wherein soluble sugars are first released, followed by their metabolism into organic acids via microbial activity.

Additionally, the combined application of cellulase and strain A30 led to a significant increase in CP content, which aligned with Rinne et al. [25]. This observation may be attributed to the lowered pH inhibited plant proteases and microbial activities, thereby preserving CP content [26]. From another perspective, the increase in CP content may also result from the concentration effect caused by fiber degradation: as fiber components such as NDF and ADF are degraded by A30 or cellulase, the relative proportion of protein in unit DM is significantly increased. However, adding cellulase or *Bacillus amyloliquefaciens* A30 exerted no significant effects on most fermentation parameters of whole-plant corn silage in this study. Zhao et al. stated that *Lactiplantibacillus plantarum* and propionic acid had no significant impact on nutrient composition or fermentation parameters in amaranth silage, which was in line with our findings [27]. Similarly, Xu et al. utilized *Lactiplantibacillus plantarum* and *Lactobacillus buchneri* to ensile whole-plant soybean–corn mixtures and found that the addition of these strains failed to improve the nutrient indices such as DM [28]. It is hypothesized that ferulic acid esters in whole-plant corn are predominantly localized in the vascular bundles of stems and the veins of leaves, while their content in grains and mesophyll tissues is extremely low. However, during the ensiling process, grains serve as the primary contributor to DM, CP, and Fat. Even if the strain A30 degrades a portion of ferulic acid esters in stems, this effect is only limited to “fiber-related dry matter” and exerts no influence on the “grain-derived DM, CP, or Fat,” which account for a much higher proportion of the total. Consequently, no significant differences were observed in the overall nutritional indices. In this study, the fermentation time (14 days) of corn bran may be insufficient to achieve stable fermentation. A shorter fermentation cycle results in inadequate microbial proliferation, which in turn leads to insufficient organic acid accumulation and a relatively high pH value.

Our results demonstrated that the addition of strain A30 significantly reduced cellulose of both corn bran and whole-plant corn silage. This reduction can be attributed to FAE produced by strain A30, which breaks the ferulic acid ester bonds in plant cell walls and thereby enhances cellulase accessibility to structural polysaccharides. This mechanism of action was further confirmed by the study reported by Li et al. [29]. They found that inoculating alfalfa silage with ferulic acid esterase-producing LAB reduced NDF and ADF content, suggesting a direct role of FAE in degrading cell wall structures. Thus, FAEs increased plant cell wall permeability, facilitating contact between hydrolytic enzymes or acids and polysaccharides, thereby promoting their hydrolysis into small-molecule monosaccharides. Meanwhile, this could be interpreted that the low pH in silage would promote acid hydrolysis of hemicellulose [30]. Dewar et al. (1963) indicated that structural carbohydrates were initially hydrolyzed by enzymes and subsequently (storage of 7–28 days) by acid hydrolysis at low pH [31]. The CEL + A30 group exhibited higher fiber degradation rate and lactic acid and acetic acid content than other treatments. This might be because the soluble sugars released from fiber degradation serve as sufficient substrates for lactic acid bacteria, promoting the synthesis of lactic acid and acetic acid. The accumulation of lactic acid and acetic acid further reduces the pH value of silage, creating an acidic environment that inhibits the activity of harmful microorganisms and minimizes nutrient loss.

In this study, cellulase and FAE activity levels exhibited a downward trend during the fermentation stage. This phenomenon was primarily attributed to the accumulation of lactic acid and acetic acid in silage, which lowered pH beyond the optimal range for enzyme activity. Additionally, microorganisms showed a strong preference for utilizing easily degradable substrates such as water-soluble carbohydrates and hemicellulose. As these substrates were depleted, the demand for cellulose degradation diminished, leading to reduced enzyme synthesis.

Microbial activity mainly drives the silage process, and the dynamics of microbial communities in the process of ensiling have a crucial impact on silage quality. Alpha diversity was employed to estimate the abundance, diversity, and evenness of species within the bacterial community [32]. In this study, significantly lower Shannon, Chao, and Ace indices (*p* < 0.05) and a higher Simpson index (*p* < 0.05) in corn bran silage and whole-plant corn silage were obtained, indicating that the microbial diversity and richness were reduced. Similarly, Wang et al. discovered a tendency toward reduced community abundance and diversity in red clover silage [33]. This phenomenon can be attributed to the inherent nature of ensiling, which involves the gradual formation of an anaerobic and acidic environment [34]. This environmental shift exerts a “directional selection” effect on the microbial community: only microorganisms adapted to low pH and anaerobic conditions (e.g., *Lactobacillus*) can survive and proliferate, while most aerobic microbes and those thriving in neutral/alkaline environments are inhibited or eliminated. Ultimately, this leads to a reduction in community diversity. Meanwhile, dominant microbial groups (particularly *Lactobacillus* and the inoculated *Bacillus amyloliquefaciens* A30) further compress the ecological niches of other microorganisms through resource competition and the inhibitory effects of their metabolic products, thereby exacerbating the decline in community diversity. Furthermore, the metabolic changes in the raw material substrates impose an additional layer of selection, favoring microorganisms with specific functional traits (e.g., fiber-degrading capacity) while reducing species with functional redundancy. This process indirectly contributes to the decreased microbial diversity.

*Delftia* can inhibit mycelial growth of the pathogen causing potato late blight and is also an essential degrading bacterium in the process of deoxynivalenol degradation [35,36]. *Acinetobacter* and *Enterobacter* are unfavorable for preserving the fermentation quality and nutrients of mixed silage [28,37]. In this study, strain A30 increased the relative abundance of *Lactobacillus*, *Delftia*, and *Bacillus,* and suppressed pathogenic genera such as *Acinetobacter*, *Enterobacter*, and *Sphingobacterium*. The interaction between *Bacillus amyloliquefaciens* and LAB enhances lactic acid production, which in turn lowers the pH value of silage and further inhibits the proliferation of harmful bacteria.

In this study, FAEs, hemicellulose, as well as cellulose showed a highly significant correlation with *Bacillus* (*p* < 0.001) in corn bran silage and whole-plant corn silage. This correlation can be ascribed to the fact that the added A30 consumes nutrients during fermentation and then produces FAEs. In addition, FAEs can hydrolyze ferulic acid ester bonds in plant cell walls, disrupting their dense spatial structure and thereby promoting substantial degradation of cellulose and hemicellulose [38]. Interestingly, lactic acid and acetic acid showed a significant positive correlation not only with *Lactobacillus* (*p* < 0.001) but also with *Bacillus* (*p* < 0.05). Bai et al. observed that separate inoculation of *Bacillus amyloliquefaciens* and *Bacillus subtilis* in whole-plant corn silage both increased lactic acid, although acetic acid was reduced in the group inoculated with *Bacillus amyloliquefaciens* [15]. We inferred that certain *Bacillus* strains may influence organic acid production during fermentation, either directly or via their metabolites, thereby promoting organic acid accumulation.

This study confirms the application value of the ferulic acid ester-producing strain A30 in corn bran and whole-plant corn silage. Notably, its fiber degradation efficiency is further enhanced when combined with cellulase. However, the present study only demonstrated excellent degradation effects through fiber-related indicators, and its potential to improve animal production performance remains unknown. In future research, optimized combinations of multiple strains or enzymes could be developed to leverage their respective advantages, thereby enhancing both the fermentation quality of silage and the production performance of animals. Additionally, in vivo degradation rate assays and feeding experiments should be conducted to further evaluate the efficacy of strain A30.

## 5. Conclusions

Adding A30 to corn bran and whole-plant corn improved their fiber degradation and increased the abundance of beneficial bacterium, while reducing the proportion of pathogenic genera. In conclusion, the supplementation of strain A30 to corn bran and whole-plant corn facilitated fiber degradation and stabilized the microecological balance.

## Figures and Tables

**Table 1:** Characteristics of the corn bran and whole-plant corn.

Items	Corn Bran	Whole-Plant Corn
DM (g/kg FW)	449.73 ± 0.42	351.12 ± 0.71
CP (g/kg DM)	145.34 ± 2.12	81.11 ± 1.37
ASP (g/kg CP)	488.37 ± 2.87	4.02 ± 0.25
Fat (g/kg DM)	51.77 ± 1.14	25.36 ± 0.63
NDF (g/kg DM)	589.48 ± 3.29	575.45 ± 2.58
ADF (g/kg DM)	292.05 ± 2.31	294.53 ± 1.13
ADL (g/kg DM)	60.55 ± 1.52	23.23 ± 0.36
Hemicellulose (g/kg DM)	316.17 ± 2.82	280.93 ± 1.74
Cellulose (g/kg DM)	231.90 ± 2.19	271.30 ± 1.43

FW, fresh weight; DM, dry matter; CP, crude protein; ASP, acid-soluble protein; Fat, crude fat; NDF, neutral detergent fiber; ADF, acid detergent fiber; ADL, acid detergent lignin.

**Table 2:** Fermentation characteristics of corn bran ensiled for 14 d.

Item	No Cellulase		Cellulase		*p*-Value		
	No A30	A30	No A30	A30	CEL	A30	A × C
	(CK)	(A30)	(CEL)	(CEL + A30)			
pH	4.28 ± 0.01 ^A,a^	4.27 ± 0.01 ^A,a^	4.28 ± 0.01 ^A,a^	4.25 ± 0.01 ^B,b^	0.076	0.001	0.021
Lactic acid (g/kg DM)	44.83 ± 0.08	46.72 ± 1.54	46.33 ± 0.45	47.71 ± 1.27	0.069	0.024	0.680
Acetic acid (g/kg DM)	5.66 ± 0.09 ^B,b^	8.06 ± 0.13 ^A,b^	6.21 ± 0.16 ^B,a^	8.62 ± 0.15 ^A,a^	<0.001	<0.001	1.000

DM, dry matter; CK, silage with no additive; CEL, silage treated with cellulase; A30, silage treated with *Bacillus amyloliquefaciens* A30; CEL + A30, silage treated with cellulase and *Bacillus amyloliquefaciens* A30. Different lowercase letters (e.g., a, b) for the same index within the same row denote statistically significant differences between different enzyme treatments under bacterial treatment; capital letters (e.g., A, B) within the same row denote significant differences between different bacterial treatments under enzyme treatment. Data are presented as the “mean ± standard deviation”.

**Table 3:** Fermentation characteristics of whole-plant corn ensiled for 60 d.

Item	No Cellulase		Cellulase		*p*-Value		
	No A30	A30	No A30	A30	CEL	A30	A × C
	(CK)	(A30)	(CEL)	(CEL + A30)			
pH	4.12 ± 0.01 ^B,a^	4.14 ± 0.01 ^A,b^	4.10 ± 0.01 ^B,b^	4.17 ± 0.01 ^A,a^	0.172	<0.001	<0.001
Lactic acid (g/kg DM)	80.67 ± 0.96	81.09 ± 1.08	82.66 ± 0.65	81.12 ± 1.45	0.142	0.397	0.152
Acetic acid (g/kg DM)	23.62 ± 0.78	23.96 ± 1.08	23.16 ± 0.95	22.91 ± 0.91	0.201	0.936	0.600

DM, dry matter; CK, silage with no additive; CEL, silage treated with cellulase; A30, silage treated with *Bacillus amyloliquefaciens* A30; CEL + A30, silage treated with cellulase and *Bacillus amyloliquefaciens* A30. Different lowercase letters (e.g., a, b) for the same index within the same row denote statistically significant differences between different enzyme treatments under bacterial treatment; capital letters (e.g., A, B) within the same row denote significant differences between different bacterial treatments under enzyme treatment. Data are presented as the “mean ± standard deviation”.

**Table 4:** Nutritional components of corn bran ensiled for 14 d.

Item	No Cellulase		Cellulase		*p*-Value		
	No A30	A30	No A30	A30	CEL	A30	A × C
	(CK)	(A30)	(CEL)	(CEL + A30)			
DM (g/kg FW)	451.88 ± 1.32 ^A,a^	450.33 ± 0.69 ^A,a^	449.15 ± 1.01 ^A,b^	449.23 ± 1.88 ^A,a^	0.034	0.357	0.308
CP (g/kg DM)	150.77 ± 2.51 ^A,a^	153.23 ± 2.30 ^A,b^	153.18 ± 0.72 ^B,a^	161.67 ± 1.58 ^A,a^	0.001	0.001	0.026
ASP (g/kg CP)	491.13 ± 1.75 ^B,b^	511.34 ± 4.33 ^A,b^	505.22 ± 1.38 ^B,a^	530.12 ± 4.14 ^A,a^	<0.001	<0.001	0.240
Fat (g/kg DM)	50.87 ± 0.60	53.02 ± 2.19	51.89 ± 1.63	53.23 ± 0.95	0.493	0.075	0.650
NDF (g/kg DM)	526.29 ± 7.45 ^A,a^	524.48 ± 2.73 ^A,a^	472.80 ± 3.06 ^A,b^	454.15 ± 2.88 ^B,b^	<0.001	0.004	0.012
ADF (g/kg DM)	235.64 ± 7.20 ^A,a^	217.80 ± 2.49 ^B,a^	202.17 ± 2.35 ^A,b^	207.25 ± 1.71 ^A,b^	<0.001	0.027	0.001
ADL (g/kg DM)	47.35 ± 1.70 ^A,b^	43.04 ± 0.64 ^A,a^	40.96 ± 4.41 ^A,a^	41.98 ± 0.13 ^A,a^	0.027	0.266	0.089
Hemicellulose (g/kg DM)	290.64 ± 4.41 ^B,a^	306.67 ± 2.78 ^A,a^	270.62 ± 2.79 ^A,b^	246.89 ± 2.77 ^B,b^	<0.001	0.076	<0.001
Cellulose (g/kg DM)	188.29 ± 6.09 ^A,a^	175.82 ± 3.03 ^B,a^	167.88 ± 2.22 ^A,b^	160.32 ± 0.66 ^B,b^	<0.001	0.001	0.271

FW, fresh weight; DM, dry matter; CP, crude protein; ASP, acid-soluble protein; Fat, crude fat; NDF, neutral detergent fiber; ADF, acid detergent fiber; ADL, acid detergent lignin. CK, silage with no additive; CEL, silage treated with cellulase; A30, silage treated with *Bacillus amyloliquefaciens* A30; CEL + A30, silage treated with cellulase and *Bacillus amyloliquefaciens* A30. Different lowercase letters (e.g., a, b) for the same index within the same row denote statistically significant differences between different enzyme treatments under bacterial treatment; capital letters (e.g., A, B) within the same row denote significant differences between different bacterial treatments under enzyme treatment. Data are presented as the “mean ± standard deviation”.

**Table 5:** Nutritional components of whole-plant corn ensiled for 60 d. (Dry matter basis).

Item	No Cellulase		Cellulase		*p*-Value		
	No A30	A30	No A30	A30	CEL	A30	A × C
	(CK)	(A30)	(CEL)	(CEL + A30)			
DM (g/kg FW)	325.60 ± 1.40	351.30 ± 1.87	353.30 ± 3.58	351.50 ± 3.09	0.774	0.340	0.873
CP (g/kg DM)	93.08 ± 2.36	92.63 ± 1.61	92.07 ± 0.91	93.02 ± 0.99	0.743	0.794	0.467
ASP (g/kg CP)	2.50 ± 0.32	2.50 ± 0.33	2.59 ± 0.31	2.49 ± 0.06	0.795	0.765	0.780
Fat (g/kg DM)	31.68 ± 1.23	32.11 ± 0.30	32.67 ± 0.78	32.51 ± 0.92	0.204	0.799	0.574
NDF (g/kg DM)	557.60 ± 1.08 ^A,a^	538.70 ± 3.25 ^B,a^	492.60 ± 1.92 ^A,b^	461.50 ± 1.50 ^B,b^	<0.001	<0.001	0.001
ADF (g/kg DM)	279.40 ± 2.06 ^A,a^	273.90 ± 1.45 ^B,a^	267.30 ± 1.13 ^A,b^	249.70 ± 1.61 ^B,b^	<0.001	<0.001	<0.001
ADL (g/kg DM)	22.24 ± 0.25 ^A,a^	21.73 ± 0.63 ^A,a^	21.91 ± 0.44 ^A,a^	21.71 ± 0.40 ^A,a^	0.240	0.464	0.990
Hemicellulose (g/kg DM)	278.20 ± 2.24 ^A,a^	264.80 ± 1.69 ^B,a^	225.30 ± 2.51 ^A,b^	211.80 ± 2.42 ^B,b^	<0.001	<0.001	0.972
Cellulose (g/kg DM)	257.16 ± 2.12 ^A,a^	251.86 ± 1.27 ^B,a^	245.40 ± 1.55 ^A,b^	227.99 ± 1.52 ^B,b^	<0.001	<0.001	<0.001

FW, fresh weight; DM, dry matter; CP, crude protein; ASP, acid-soluble protein; Fat, crude fat; NDF, neutral detergent fiber; ADF, acid detergent fiber; ADL, acid detergent lignin. CK, silage with no additive; CEL, silage treated with cellulase; A30, silage treated with *Bacillus amyloliquefaciens* A30; CEL + A30, silage treated with cellulase and *Bacillus amyloliquefaciens* A30. Different lowercase letters (e.g., a, b) for the same index within the same row denote statistically significant differences between different enzyme treatments under bacterial treatment; capital letters (e.g., A, B) within the same row denote significant differences between different bacterial treatments under enzyme treatment. Data are presented as the “mean ± standard deviation”.

**Table 6:** Fiber degradation of corn bran silage and whole-plant corn silage.

Item	Treatments	Degradation Ratio (%)	
		Corn Bran Silage	Whole-Plant Corn Silage
NDF	CK	10.72 ± 0.31 ^c^	3.10 ± 0.25 ^d^
	CEL	19.79 ± 0.12 ^b^	14.40 ± 0.22 ^b^
	A30	11.03 ± 0.17 ^c^	6.39 ± 0.41 ^c^
	CEL + A30	22.96 ± 0.12 ^a^	19.80 ± 0.39 ^a^
ADF	CK	19.32 ± 0.33 ^c^	5.14 ± 0.32 ^c^
	CEL	30.78 ± 0.52 ^a^	9.24 ± 0.57 ^b^
	A30	25.42 ± 0.41 ^b^	7.01 ± 0.41 ^c^
	CEL + A30	29.04 ± 0.47 ^a^	15.22 ± 0.30 ^a^
ADL	CK	21.79 ± 1.21 ^b^	4.26 ± 0.38 ^b^
	CEL	32.35 ± 2.15 ^a^	5.70 ± 0.20 ^a^
	A30	28.92 ± 2.33 ^a^	5.14 ± 0.34 ^a^
	CEL + A30	30.66 ± 2.60 ^a^	6.54 ± 0.29 ^a^
Hemicellulose	CK	8.07 ± 1.72 ^c^	0.97 ± 0.84 ^d^
	CEL	14.41 ± 0.94 ^b^	19.80 ± 1.03 ^b^
	A30	3.00 ± 0.75 ^d^	5.74 ± 0.74 ^c^
	CEL + A30	21.91 ± 1.66 ^a^	24.61 ± 1.14 ^a^
Cellulose	CK	18.81 ± 0.64 ^d^	5.21 ± 0.27 ^d^
	CEL	27.61 ± 0.53 ^b^	9.55 ± 0.35 ^b^
	A30	24.18 ± 0.68 ^c^	7.17 ± 0.30 ^c^
	CEL + A30	30.87 ± 0.37 ^a^	15.96 ± 0.21 ^a^

NDF, neutral detergent fiber; ADF, acid detergent fiber; ADL, acid detergent lignin. CK, silage with no additive; CEL, silage treated with cellulase; A30, silage treated with *Bacillus amyloliquefaciens* A30; CEL + A30, silage treated with cellulase and *Bacillus amyloliquefaciens* A30. Different lowercase letters (e.g., a, b) for the same index within the same column denote significant differences between groups.

**Table 7:** Microorganism quantity of corn bran ensiled for 14 d.

Item	No Cellulase		Cellulase		*p*-Value		
	No A30	A30	No A30	A30	CEL	A30	A × C
	(CK)	(A30)	(CEL)	(CEL + A30)			
Bacteria lg(CFU/g)	8.14 ± 0.09	8.31 ± 0.08	8.11 ± 0.25	8.31 ± 0.20	0.895	0.099	0.869
LAB lg(CFU/g)	8.46 ± 0.14	8.34 ± 0.10	8.45 ± 0.21	8.56 ± 0.15	0.278	0.957	0.264
Mold lg(CFU/g)	4.28 ± 0.09	4.32 ± 0.13	4.18 ± 0.11	4.28 ± 0.06	0.272	0.251	0.678

LAB, lactic acid bacteria. CK, silage with no additive; CEL, silage treated with cellulase; A30, silage treated with *Bacillus amyloliquefaciens* A30; CEL + A30, silage treated with cellulase and *Bacillus amyloliquefaciens* A30.

**Table 8:** Microorganism quantity of whole-plant corn ensiled for 60 d.

Item	No Cellulase		Cellulase		*p*-value		
	No A30	A30	No A30	A30	CEL	A30	A × C
	(CK)	(A30)	(CEL)	(CEL + A30)			
Bacteria lg(CFU/g)	5.90 ± 0.09 ^B^	6.41 ± 0.08 ^A^	6.00 ± 0.16 ^B^	6.49 ± 0.18 ^A^	0.284	<0.001	0.883
LAB lg(CFU/g)	8.86 ± 0.13	8.85 ± 0.14	8.88 ± 0.21	8.89 ± 0.16	0.744	0.986	0.959
Mold lg(CFU/g)	3.40 ± 0.15	3.50 ± 0.10	3.45 ± 0.10	3.40 ± 0.08	0.767	0.694	0.275

LAB, lactic acid bacteria. CK, silage with no additive; CEL, silage treated with cellulase; A30, silage treated with *Bacillus amyloliquefaciens* A30; CEL + A30, silage treated with cellulase and *Bacillus amyloliquefaciens* A30. Different capital letters (e.g., A, B) within the same row denote significant differences between different bacterial treatments under enzyme treatment. Data are presented as the “mean ± standard deviation”.

**Table 9:** Alpha diversity of bacteria in corn bran silage.

Day	Treatments	Shannon	Simpson	Chao	Ace	Coverage
0 d	CK	3.26	0.13	384.51	369.36	0.9986
	CEL	3.01	0.12	374.91	361.51	0.9987
	A30	3.02	0.18	377.76	364.90	0.9987
	CEL + A30	2.94	0.19	370.71	357.90	0.9986
14 d	CK	2.42 ^a^	0.31 ^b^	314.22 ^a^	323.17 ^a^	0.9987
	CEL	2.74 ^a^	0.26 ^b^	322.25 ^a^	329.53 ^a^	0.9988
	A30	1.87 ^b^	0.68 ^a^	265.86 ^b^	264.01 ^b^	0.9987
	CEL + A30	1.90 ^b^	0.59 ^a^	268.79 ^b^	265.64 ^b^	0.9985

CK, silage with no additive; CEL, silage treated with cellulase; A30, silage treated with *Bacillus amyloliquefaciens* A30; CEL + A30, silage treated with cellulase and *Bacillus amyloliquefaciens* A30. Different lowercase letters (e.g., a, b) for the same index within the same row denote significant differences between groups at the same fermentation time.

**Table 10:** Alpha diversity of bacteria in whole-plant corn silage.

Day	Treatments	Shannon	Simpson	Chao	Ace	Coverage
0 d	CK	3.52	0.09	775.6	741.6	0.9969
	CEL	3.48	0.07	778.0	774.2	0.9977
	A30	3.58	0.07	784.6	737.1	0.9970
	CEL + A30	3.60	0.06	783.0	750.5	0.9975
60 d	CK	2.14 ^a^	0.25 ^b^	478.0 ^a^	437.9 ^a^	0.9975
	CEL	2.17 ^a^	0.23 ^b^	485.2 ^a^	426.4 ^a^	0.9976
	A30	2.05 ^b^	0.34 ^a^	430.7 ^b^	404.3 ^b^	0.9975
	CEL + A30	2.02 ^b^	0.35 ^a^	424.1 ^b^	403.7 ^b^	0.9973

CK, silage with no additive; CEL, silage treated with cellulase; A30, silage treated with *Bacillus amyloliquefaciens* A30; CEL + A30, silage treated with cellulase and *Bacillus amyloliquefaciens* A30. Different lowercase letters (e.g., a, b) for the same index within the same row denote significant differences between groups at the same fermentation time.

## Data Availability

The raw data supporting the conclusions of this article will be made available by the authors on request.

## Abbreviations

The following abbreviations are used in this manuscript:

FM	Fresh weight
DM	Dry matter
CP	Crude protein
ASP	Acid-soluble protein
Fat	Crude fat
LAB	Lactic acid bacteria
NDF	Neutral detergent fiber
ADF	Acid detergent fiber
ADL	Acid detergent lignin
CEL	Cellulase
A30	*Bacillus amyloliquefaciens strain* A30
CEL + A30	Cellulase and *Bacillus amyloliquefaciens strain* A30
FAE	Ferulic acid esterase
